# Roles of Protein Ubiquitination and Degradation Kinetics in Biological Oscillations

**DOI:** 10.1371/journal.pone.0034616

**Published:** 2012-04-10

**Authors:** Lida Xu, Zhilin Qu

**Affiliations:** 1 Department of Medicine (Cardiology), David Geffen School of Medicine, University of California Los Angeles, Los Angeles, California, United States of America; 2 Department of Systems Science, Beijing Normal University, Beijing, People's Republic of China; University of Leeds, United Kingdom

## Abstract

Protein ubiquitination and degradation play important roles in many biological functions and are associated with many human diseases. It is well known that for biochemical oscillations to occur, proper degradation rates of the participating proteins are needed. In most mathematical models of biochemical reactions, linear degradation kinetics has been used. However, the degradation kinetics in real systems may be nonlinear, and how nonlinear degradation kinetics affects biological oscillations are not well understood. In this study, we first develop a biochemical reaction model of protein ubiquitination and degradation and calculate the degradation rate against the concentration of the free substrate. We show that the protein degradation kinetics mainly follows the Michaelis-Menten formulation with a time delay caused by ubiquitination and deubiquitination. We then study analytically how the Michaelis-Menten degradation kinetics affects the instabilities that lead to oscillations using three generic oscillation models: 1) a positive feedback mediated oscillator; 2) a positive-plus-negative feedback mediated oscillator; and 3) a negative feedback mediated oscillator. In all three cases, nonlinear degradation kinetics promotes oscillations, especially for the negative feedback mediated oscillator, resulting in much larger oscillation amplitudes and slower frequencies than those observed with linear kinetics. However, the time delay due to protein ubiquitination and deubiquitination generally suppresses oscillations, reducing the amplitude and increasing the frequency of the oscillations. These theoretical analyses provide mechanistic insights into the effects of specific proteins in the ubiquitination-proteasome system on biological oscillations.

## Introduction

Protein ubiquitination and degradation, regulated by the ubiquitin-proteasome system (UPS), play important roles in many fundamental biological functions and are associated with many human diseases [1], [2], [3], [4]. For a given protein synthesis rate, a proper degradation rate is needed to maintain absolute protein abundance and thereby normal biological functions. For example, in the mammalian cell cycle, cyclins must be properly degraded for normal cell cycle control [5]: failure to ubiquitinate and degrade cyclin B due to deletion of cdc20 (of the E3 ligase anaphase promoting complex/cyclosome (APC) leads to cyclin B accumulation and causes M-phase arrest [6], and failure to ubiquitinate and degrade cyclin E due to deletion of cul1 or skp2 of the E3 ligase SCF (Skp1/Cul1/F-box protein) promotes cyclin E accumulation and endoreduplication [7], [8]. In circadian rhythms, it has been shown that a mutation of the F-box protein Fbxl3, which mediates degradation of cryptochrome proteins, lengthens the period of the circadian clock [9], [10]. Despite the well known roles of protein degradation in maintaining protein homeostasis and thereby biological oscillations, how protein ubiquitination and degradation kinetics affects protein network dynamics is not well understood.

In many mathematical models of biochemical reactions [11], [12], [13], [14], [15], the degradation rate of a substrate protein S has been modeled as being linearly proportional to its concentration [S], i.e., 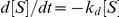. This implies that the protein content decays exponentially (i.e., 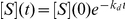), which has been shown in experimental measurements [2], [16]. However, other experiments [17], [18] showed linear decays, indicating that the protein is degraded at a constant rate. One possible explanation for this discrepancy is that the degradation rate follows a Michaelis-Menten (MM) function, i.e., 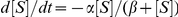. When the dissociation constant β is much smaller than [*S*], i.e., 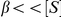, then 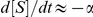, and thus the degradation rate is close to the maximum rate α, which is a constant. When 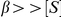, 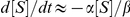, and thus the degradation kinetics is almost linear. The MM kinetics for protein degradation was also used in mathematical modeling studies [14], [19], [20], [21], mainly following the Goldbeter-Koshland formulation [22]. In a recent study, Wong et al [18] showed in a mathematical model of a synthetic circuit of E. Coli that the MM degradation kinetics significantly enlarges the parameter space for oscillations, however, the underlying mechanisms remain unclear.

Therefore, this raises several questions in the perspective of mathematical modeling and nonlinear dynamics of biochemical reactions: 1) what is the kinetics of protein degradation? 2) how do protein degradation and its kinetics affect the dynamics of a biochemical system besides its role in maintaining a proper protein abundance? 3) how does a specific protein in the UPS affect the dynamics of a biochemical system. To address the first question, we developed a detailed mathematical model of the UPS based on existing information and a recent experimentally-based model [23]. Using this model, we studied the protein degradation kinetics and showed that they are mainly MM kinetics with time delays (due to ubiquitination and deubiquitination). Since the detailed model is too complex to be used for a general analysis of the effects of the degradation kinetics on nonlinear dynamics, to address the second question, we used both linear and MM degradation kinetics with time delays in simplified biochemical reaction models that generate oscillations following the three typical mechanisms: 1) positive feedback; 2) positive-plus-negative feedback; and 3) negative feedback. We performed pure theoretical analyses of these models. We showed that the MM degradation kinetics enlarges the oscillatory region in all three mechanisms of oscillation, especially for the negative feedback mediated oscillations. However, the time delay in the UPS tends to stabilize the steady state, suppressing oscillations, but can turn simple oscillations into complex ones. To address the third question, we combined the detailed model of protein ubiquitination and degradation to the simplified models. We used computer simulations of these models and altered the protein concentrations in the UPS to study their effects on oscillations and explained how they affect the oscillatory dynamics based on the theoretical predictions of the simplified systems.

## Results

### Kinetics of protein degradation

**Figure 1:**
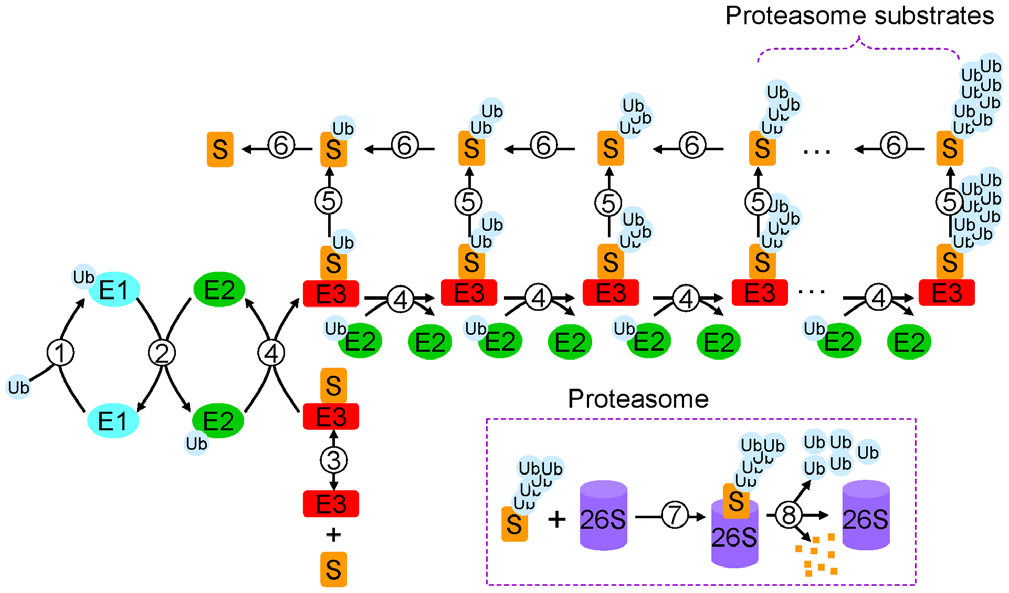
Schematic plot of the reactions in the ubiquitination and degradation model (see text for details).

In a recent study [23], Pierce et al established an assay capable of simultaneously monitoring the concentrations of substrate and its different ubiquitinated product intermediates, and their time-dependent changes. They showed that the ubiquitination of a substrate protein occurs primarily by sequential transfers of single ubiquitin molecules to the substrate. Using these experiments, they could develop a quantitative model of UPS and estimate the corresponding rate constants. In this study, we developed a mathematical model of UPS primarily based on the model by Pierce et al [23]. Figure 1 is a schematic diagram of the detailed reactions in the UPS model. Reaction step 1: Ubiquitin (Ub) is activated by the ubiquitin-activating enzyme E1. Step 2: Ub is transferred from E1 to ubiquitin-conjucating enzyme E2. Step 3: Substrate (S) binds with E3 ligase. Step 4: Ub is transferred from E2 to substrate S, forming polyubiquitin chains. Step 5: Ubiquitinated substrates dissociate with E3; Step 6: Ub dissociates with substrates (deubiquitination); Step 7: Ubiquitinated substrates with Ub chains longer than 4 bind with 26S proteasome for degradation. Step 8: Degradation of substrate S. The model equations were formulated following the law of mass action, with the differential equations and control parameter shown in Table 1. The control parameter set and the protein concentrations are similar to the ones in Pierce et al [23].

**Table 1:** Details of the UPS model.

**1. Differential equations:**
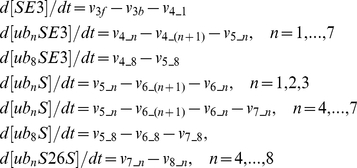
**2. Reaction rates:**
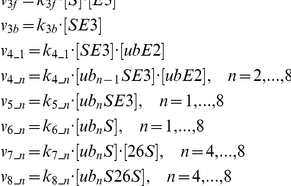
**3. Notions:**
[S]—concentration of of substrate protein; [E1]—concentration of E1; [E2]—concentration of E2; [E3]—concentration of E3; [26S]—concentration of 26S; [ubE1]—concentration of ub-E1 complex; [ubE2]—concentration of ub-E2 complex; [SE3]—concentration of S-E3 complex; [ub_n_SE3]—concentration of ub-S-E3 complex with ubiquitin chain of length n (n=1,…,8); [ub_n_S]—concentration of ub-S complex with ubiquitin chain of length n (n=1,…,8); [ub_n_S26S]—concentration of ub-S-26S complex with ubiquitin chain of length n (n=1,…,8).
**4. Parameters:**
[ub]_T_=150 nM, [E1]_T_=1,000 nM, [E2]_T_=10,000 nM, [E3]_T_=150 nM, [26S]_T_=500 nM; *k*_1f_=0.00001 (nM s)^−1^, *k*_1b_=0.55 s^−1^, *k*_2f_=0.00001 (nM s)^−1^, *k*_2b_=0.00019 (nM s)^−1^, *k*_3f_=0.001 (nM s)^−1^, *k*_3b_=0.37 s^−1^; *k*_4_1_=0.00034 (nM s)^−1^, *k*_4_2_=0.0078 (nM s)^−1^, *k*_4_3_=0.002 (nM s)^−1^, *k*_4_4_=0.0011 (nM s)^−1^, *k*_4_5_=0.00062 (nM s)^−1^, *k*_4_6_=0.00082 (nM s)^−1^, *k*_4_7_=0.0008 (nM s)^−1^, *k*_4_8_=0.0005 (nM s)^−1^; *k*_5_1_=0.4 s^−1^, *k*_5_2_=0.29 s^−1^, *k*_5_3_=0.27 s^−1^, *k*_5_4_=0.29 s^−1^, *k*_5_5_=0.89 s^−1^, *k*_5_6_=0.8 s^−1^, *k*_5_7_=0.5 s^−1^, *k*_5_8_=0.2 s^−1^; *k*_6_n_=0.05 s^−1^ (n=1, …, 8); *k*_7_4_=0.01 (nM s)^−1^, *k*_7_5_=0.02 (nM s)^−1^, *k*_7_6_=0.04 (nM s)^−1^, *k*_7_7_=0.06 (nM s)^−1^, *k*_7_8_=0.08 (nM s)^−1^; *k*_8_4_=0.1 s^−1^, *k*_8_5_=0.2 s^−1^, *k*_8_6_=0.4 s^−1^, *k*_8_7_=0.6 s^−1^, *k*_8_8_=0.8 s^−1^.

**Figure 2:**
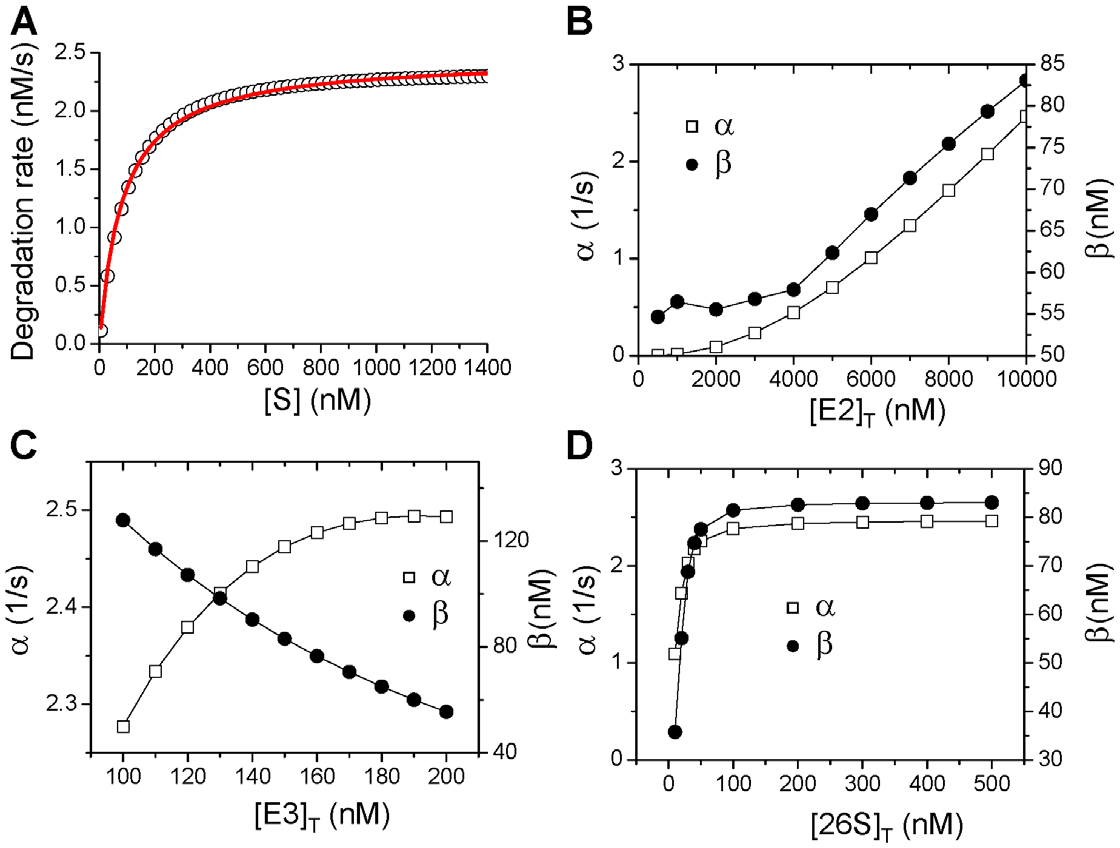
Effects of different UPS proteins on protein degradation kinetics. **A**. Degradation rate versus free substrate concentration [S] for the control parameters shown in Table 1. Symbols are calculated from the model and the line is a least square fit by the MM function: *g*([S])=*α*[S]/(*β*+[S]) with *α*=2.46 s^−1^, and *β*=83.05 nM. **B**. *α* and *β* as versus [E2]_T_. **C**. *α* and *β* versus [E3]_T_. **D**. *α* and *β* versus [26S]_T_.

For the control parameter set, the degradation rate versus the substrate concentration ([S]) can be well fit with a MM function: 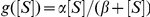 (Fig. 2A). To show how different proteins in the UPS affect the degradation kinetics, we plot the maximum degradation rate *α* and the dissociation constant *β* versus the total E2 concentration [E2]_T_ (Fig. 2B), the total E3 concentration [E3]_T_ (Fig. 2C), and the total 26S concentration [26S]_T_ (Fig. 2D). Both *α* and *β* increase as [E2]_T_ increases; *α* increases and *β* decreases as [E3]_T_ increases; both *α* and *β* increase but then saturate as [26S]_T_ increases. These observations can be understood as follows based on the reaction scheme shown in Fig. 1: 1) increasing [E2] gives rise to a faster ubiquitnation speed of S and thus a higher degradation rate, however, speeding up the ubiquitination of S reduces the free E3 so that less S-E3 complex can be formed, and thus the dissociation constant *β* increases; 2) increasing [E3] increases the ubiquitinated S and thus increases the degradation rate. More E3 speeds up the binding rate of S and E3, and thus reduces *β*; 3) as for the case of changing 26S, it is not as obvious as in the former two cases. One would expect that as 26S increases, the degradation rate increases but in fact saturates in our simulations. The explanation is that the [E2] and [E3] are not high enough to produce enough ubiquinated substrates and thus the degradation rate is insensitive to high [26S].

**Figure 3:**
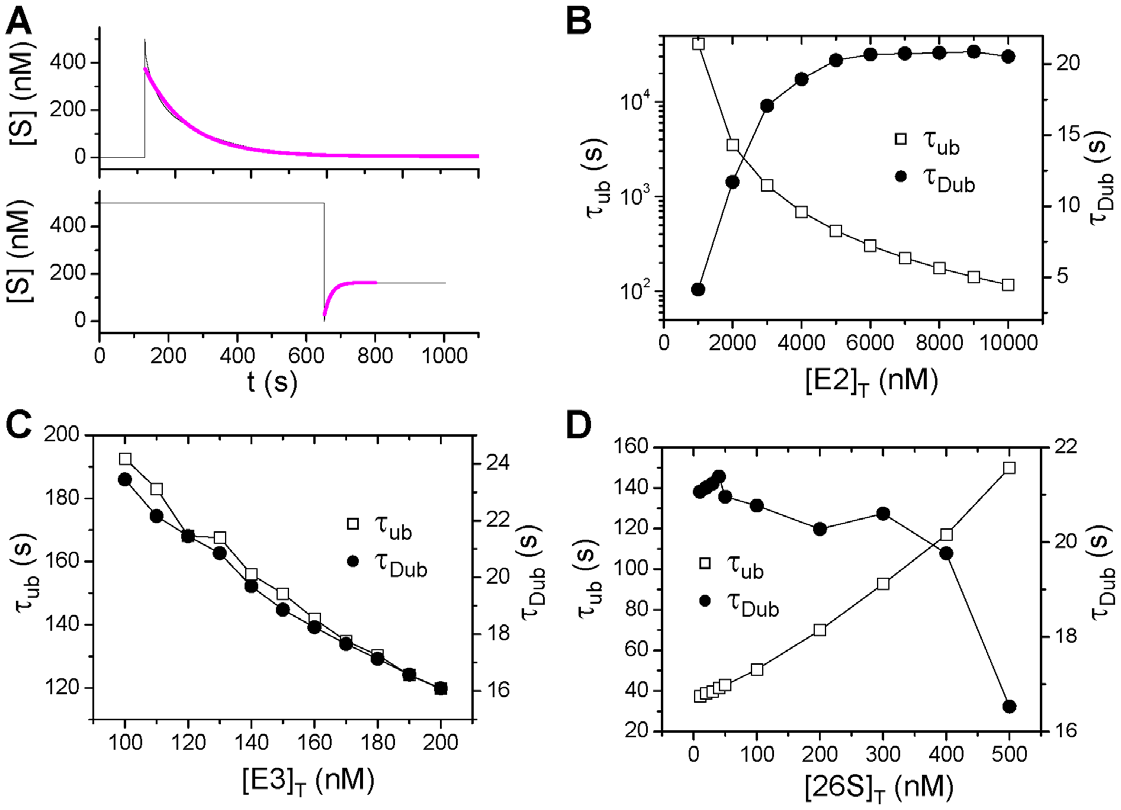
Time delay of ubiquitination and deubiquitination in the UPS. **A**. A simulation protocol of determining *τ*_ub_ in which the free [S] is switched from zero to 500 nM, and then [S] decays due to ubiquitination. The decaying curve is fitted to an exponential function (color line) to determine *τ*_ub_. **B**. A simulation protocol in determining *τ*_Dub_ in which the free [S] is held at 500 nM for 650 s for the system to equilibrium and then switched to zero. [S] then grows from zero to reach a new equilibrium state due to deubiquitination. The growth curve is fitted with an exponential function (color line) to determine *τ*_Dub_. In determining *τ*_ub_ and *τ*_Dub_, the reaction step 8 in Fig. 1 is removed to exclude the effects of degradation. **C**. *τ*_ub_ and *τ*_Dub_ versus [E3]_T_. **D**. *τ*_ub_ and *τ*_Dub_ versus [E3]_T_.

We calculated the time constants of ubiquitination and deubiquitination of the model by using the simulation protocols shown in Fig. 3A. We removed the degradation reaction (Step 8 in Fig. 1) from the model to maintain the total substrate [S] constant. To measure the ubiquitination time constant, we switch the free substrate [S] from zero to a certain value (500 nM) and fit the decaying trace with an exponential function (Fig. 3A). For the time constant of deubiquitination, we first let the system equilibrate by holding the free S at a constant (500 nM) for a certain time period and then switch the free substrate S to zero. The deubiquitination time constant is obtained by fitting the growing trace of S with an exponential function (Fig. 3A). Figure 3B plots the ubiquitination time constant (*τ*_ub_) and the deubiquination time constant (*τ*_Dub_) versus [E2]_T_, showing that *τ*_ub_ decreases and *τ*_Dub_ increases as [E2]_T_ increases. Figure 3C plots *τ*_ub_ and *τ*_Dub_ versus [E3]_T_, showing that both *τ*_ub_ and *τ*_Dub_ decreases as [E3]_T_ increases. Figure 3D plots *τ*_ub_ and *τ*_Dub_ versus [26S]_T_, showing that *τ*_ub_ increases but *τ*_Dub_ decreases slightly as [26S]_T_ increases.

### Effects of the protein ubiquitination and degradation kinetics on biochemical oscillations

To analyze in general the effects of the protein ubiquitination and degradation kinetics on biochemical oscillations, we used three typical simplified biochemical reaction circuits that can cause oscillations [24], [25]: 1) a single positive feedback loop; 2) a positive feedback loop plus a negative feedback loop; and 3) a single negative feedback loop. We then use either the linear degradation kinetics: 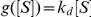 or the MM one: 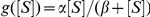 in the three biochemical reaction circuits to compare how they affect the oscillations. However, changing the degradation kinetics changes the system, therefore, for an unbiased comparison, we apply the following constraint in which the steady state (*ξ*) of the substrate protein maintains the same under the two different degradation kinetics,, which can be satisfied by requiring:
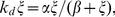
(1)leading to the following relationship:
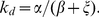
(2)In the following sections, we compare the effects of the two degradation kinetics on the stability of the steady state for the three mechanisms of oscillations, and show how time delay in degradation affects the oscillations.

**Figure 4:**
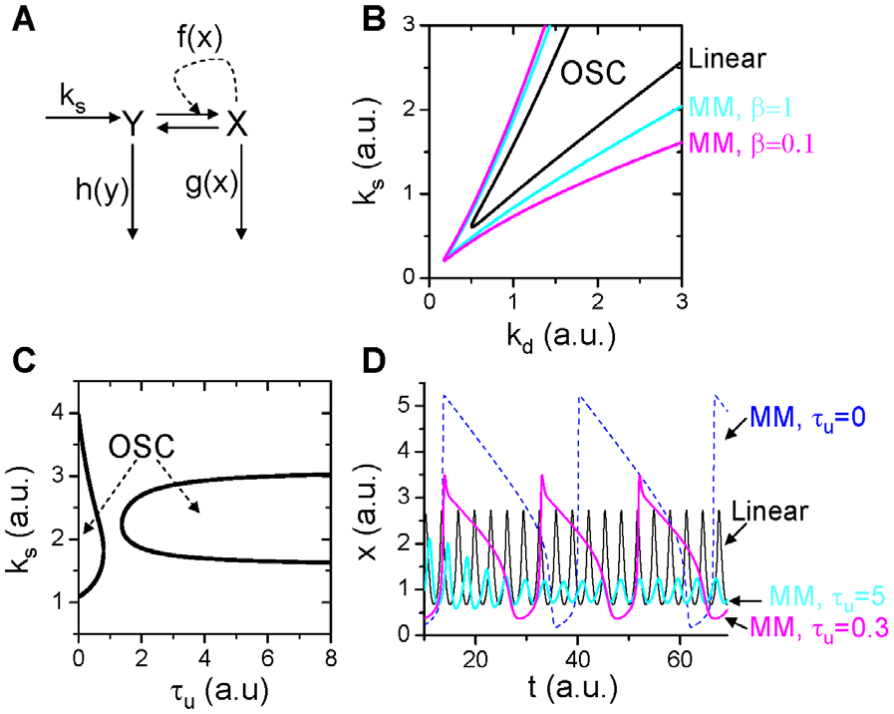
Effects of protein degradation kinetics on oscillations in positive feedback mediated oscillations. **A**. Schematic plot of the positive-feedback. **B**. Oscillation regions (marked by “OSC") under different degradation kinetic conditions. The OSC regions were obtained using the constraint of Eq.1 or Eq.2. Specifically, we first use the linear kinetics 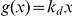 to determine the OSC region and the steady state *ξ* in the *k*_d_-*k*_s_ space. We then use the MM kinetics 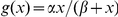 and use Eq.2 and *k*_d_ and *ξ* from the case of linear kinetics to determine *α* for different *β*, i.e., 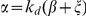. By applying this constraint, we map the OSC regions of the MM kinetics to the *k*_d_-*k*_s_ space of the case of linear kinetics so that we can compare their effects on stability fairly. **C**. The OSC region in *τ*_u_-*k*_s_ space for MM kinetics with *β*=0.1 and *k*_d_=1.7. **D**. Sample traces of *x* under different degradation kinetic conditions. *k*_s_=2.3, *k*_d_=1.7, and *ξ*=1.35 for linear degradation kinetics. *k*_s_=2.3, *ξ*=1.35, *β*=0.1, and 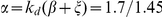 for the MM degradation kinetics. No protein Y degradation in B–D, i.e., *h*(*y*)=0. *k*_1_=0.5, *k*_2_=3.5, and *f*(*x*)=*x* ^2^.

*Positive feedback*. Positive feedback is involved in many biological processes [26], [27], [28], [29], such as glycolytic oscillation, circadian rhythm, cell cycle control, differentiation, and gene transcription. Oscillations and bistability can be caused by a single positive feedback loop in a simplified two protein reaction system (Fig. 4A), in which protein Y is synthesized, and then coverts to protein X through an autocatalytic reaction. Both protein X and Y are degraded though the UPS. The differential equations for this simple model can be written as,
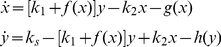
(3)where *x* and *y* are the concentrations of the two proteins. *f*(*x*) is a nonlinear function describing the strength of the positive feedback, increasing with *x*. *g*(*x*) and *h*(*y*) are the degradation rates of protein X and Y, also increasing with *x* and *y*, respectively. The trace (*Tr*) and determinant (Δ) of the Jacobian matrix for the steady state of Eq.1 are [30]:
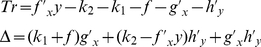
(4)where 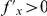, 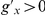 and 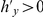 are the corresponding derivatives at the steady state, and the eigenvalues of the Jacobian is 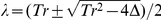. The stability criterion for a Hopf bifurcation is that the real part of *λ* changes its sign from negative to positive, which is equivalent to that Δ>0 and *Tr* changes its sign from negative to positive. Therefore, increasing 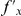 and/or reducing 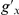 or 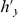 may cause *Tr* to change its sign from negative to positive to promote the Hopf bifurcation. The steady state can also become unstable via a saddle-node bifurcation which occurs when Δ changes its sign from positive to negative [30]. Here we discuss two special conditions:

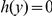
, i.e., no protein Y degradation. Under this condition, 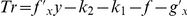 and 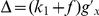. For the MM kinetics, 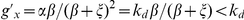 is always satisfied. Therefore, for the same degradation rate, *Tr* is larger for the MM kinetics than for the linear kinetics, and thus the steady state of the system with the linear degradation kinetics is more stable than that with the MM kinetics. Note that the determinant Δ of the Jacobian is always positive, i.e., 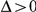 for any positive 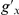, no saddle-node bifurcation can occur. Figure 4B shows the unstable regions for the linear kinetics and for the MM kinetics with different *β*, showing that the unstable region is larger for the MM kinetics and for smaller *β*.

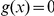
, i.e., no protein X degradation. Under this condition, 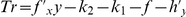 and 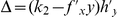. This same conclusion that the MM kinetics promotes Hopf bifurcation still holds since 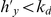 holds under the assumption that the steady states are held the same for the two degradation kinetics. In this case, since Δ can change sign, a saddle-node bifurcation can occur, but the degradation kinetics has no effects on this bifurcation since changing 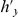 does not affect the sign of Δ.

In Eq.3, the protein degradation is instantaneous. To study the effects of the time delay that occurs due to ubiquitination and deubiquitination in the UPS, we used a simple differential equation to describe this time delay. For example, for Case i in which only protein X degradation occurs, the following equation is used to model the time delay:
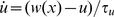
(5)We then substitute 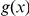 in Eq.3 by *ux*. When 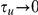, 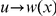, therefore, for the linear kinetics 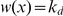 and for the MM kinetics 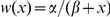, and the system recovers to Case i. One can show analytically that when *w*(*x*) is a constant (e.g., 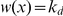), the time delay has no effect on the stability of the steady state. However, when *w*(*x*) is a function of *x*, it can alter the stability of the steady state. Figure 4C shows that as *τ*_u_ increases, the instability is first suppressed and then increased again, but remains unchanged for large *τ*_u_.

The protein degradation kinetics affects not only the stability of the steady state but also the oscillation frequency and amplitude, as expected. Figure 4D shows *x* versus time during oscillations under different conditions in which the parameters were chosen such that the steady state is maintained the same. The MM degradation kinetics results in much larger and slower oscillations than are observed with linear kinetics. The time delay of protein degradation suppresses the amplitude but increases the frequency of the oscillations.

#### Positive-plus-negative feedback

**Figure 5:**
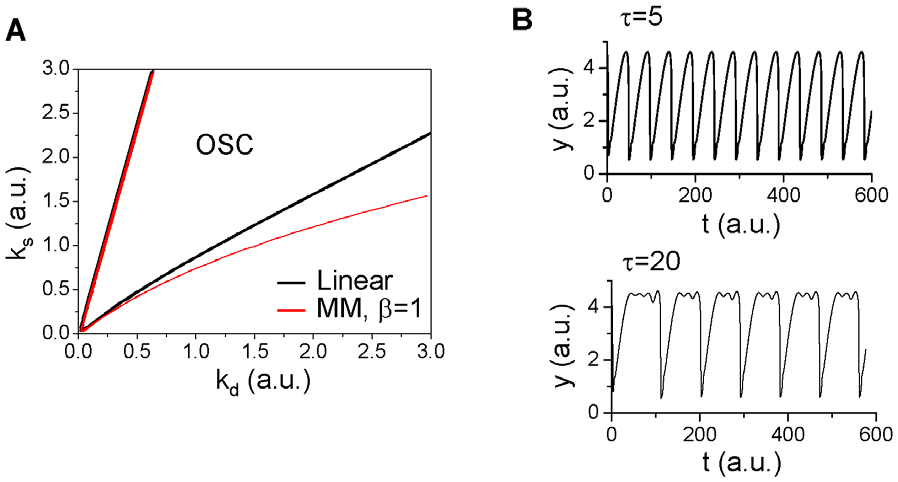
Effects of protein degradation kinetics on oscillations in positive-plus-negative feedback mediated oscillations. **A**. Oscillation regions (marked by “OSC") under different degradation kinetic conditions. The OSC regions were obtained using Eq.1 or Eq.2 to determine α in the same way as in Fig. 4. **B**. *x* versus time for two different delay time *τ*_u_. The time delay was simulated by 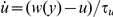 and h(y) in Eq.6 was substituted by *uy*. 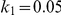, 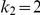, 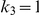, 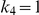, 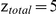, and *f*(*x*)=*x* ^2^.

The combination of a positive feedback loop and negative feedback loop can give rise to many complex behaviors [12], [31], [32], [33], [34]. In many biological systems, a fast positive feedback loop causes a steep sigmoidal or bistable response, while a delayed negative feedback makes the system oscillate. This is the most common mechanism of oscillations in biological systems [25], [31], [35], [36]. For example, in cell cycle control [12], [37], [38], the cyclin-CDK complex is activated by CDK phosphorylation, which in turn leads to further autocatalytic CDK phosphorylation, forming a *positive* feedback loop that gives rise to the bistability of CDK activity. Active cyclin-CDK then activates F-box protein to activate the SCF E3 ligase or CDC20/CDH1 to activate the APC E3 ligase causing degradation of the unbound cyclin, thereby forming a *negative* feedback loop. The minimum model that can describe this combined positive-negative feedback is given by the following differential equations:
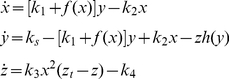
(6)where *z* is the protein that facilitates protein Y degradation and is activated by protein x with a time delay. When *z* is constant, Eq.6 becomes Eq.3 with *g*(*x*)=0. Although the degradation kinetics has no effect on the saddle-node bifurcation, it still affects the oscillations (Fig. 5A). In addition, the time delay causes more complex oscillations. Figure 5B shows two recordings from Eq.6 for two different time delays, when *τ*_u_=5, the oscillations are regular, but when *τ*_u_=20, the oscillations become complex, small amplitude oscillations occur alternatively with large amplitude oscillations. The frequency of the small amplitude oscillations is similar to the regular oscillations but the large amplitude oscillations occur at a slower frequency.

#### Negative feedback

**Figure 6:**
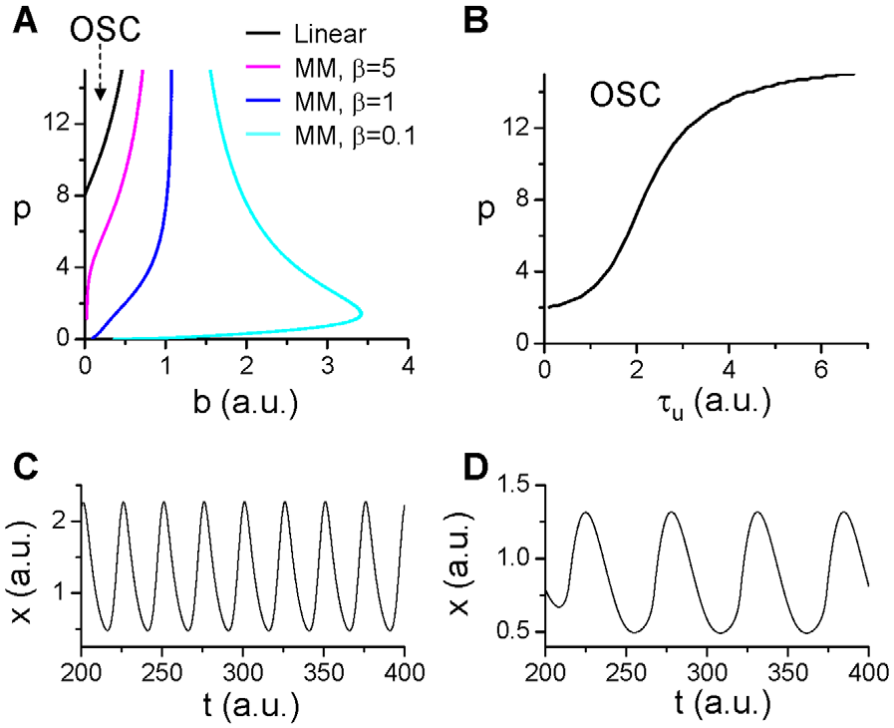
Effects of protein degradation kinetics on oscillations in negative feedback mediated oscillations. **A**. The oscillation region (marked by “OSC") under different degradation kinetic conditions. The OSC regions were obtained using the constraint of Eq.1 or Eq.2. Specifically, we first use the linear kinetics 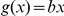 (and 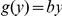, 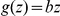) to determine the OSC region and the steady state *ξ* in the *b*-*p* space. We then use the MM kinetics 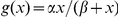 (and 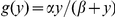, 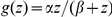) and use Eq.2 (*k*_d_=*b*) and b and *ξ* from the case of linear kinetics to determine *α* for different *β*, i.e., 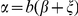. By applying this constraint, we map the OSC regions of the MM kinetics to the *b*-*p* space of the case of linear kinetics so that we can compare their effects on stability fairly. **B**. The OSC region in *τ*_u_-*p* space for *β*=1, *b*=0.5). **C**. *x* versus time for linear degradation kinetics with *p*=12 and *b*=0.15. **D**. *x* versus time for MM kinetics with *p*=0.1, *β*=0.1, and *b*=0.5.

Oscillations can be caused by a single negative feedback loop which was first proposed by Goodwin [39], [40]. Here we use a simplified version with the following equations [24]:
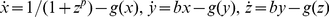
(7)where *p* and *b* are parameters, and *g*(*x*), *g*(*y*) and *g*(*z*) are the degradation rates. When 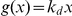, 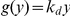, and 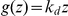 with 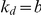, then the steady state is 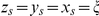 with *ξ* determined by the equation: 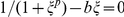. Linear stability analysis of the steady state gives rise to the following eigenvalues [see Ref. [24] for detailed analysis]:
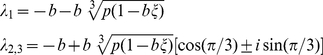
(8)Since 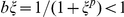, *λ*_1_<0 always satisfies, and the steady state is unstable when Re(*λ*_2,3_)>0, i.e., 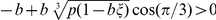, which leads to 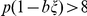. Since 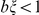, *p*>8 is required for a Hopf bifurcation to occur leading to oscillations (See Fig. 6A). In other words, for oscillations to occur, a very high cooperativity of the negative feedback kinetics is required.

When 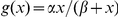, 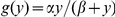, and 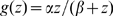, and one also assumes 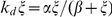 with 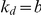, then the eigenvalues of the Jacobian of the steady state *ξ* of Eq.8 become:
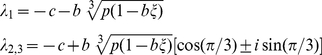
(9)where 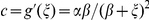 is the slope of the degradation kinetics. Hopf bifurcation occurs when 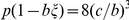. Therefore, as long as 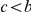, which is always satisfied under the condition of the same degradation rate, oscillations are promoted.

In Fig. 6A, we plot the boundaries for stability in b-p parameter space for different degradation kinetics, showing that the oscillation region is greatly enlarged by the MM kinetics especially for small *β*. Note that the MM kinetics dramatically reduces the cooperativity coefficient p needed for oscillations, i.e., oscillations can even occur for *p*<<1 when *β*<<1. The time delay of degradation causes stabilization of the system (Fig. 6B). Figures 6 C and D show two examples of oscillations for the linear and the MM degradation kinetics, respectively.

The problem that a high cooperatitivty is needed for oscillations to occur in the Goodwin model was solved by Bliss et al [24], [41] who showed that by changing *g*(*z*) from the original linear function to a MM function, oscillations can occur in the model for *p*=1. Here we show that protein degradation follows the MM kinetics and therefore high cooperativity of the negative feedback is not necessary for promoting negative feedback mediated oscillations in biochemical reaction networks.

### Effects of the UPS proteins on oscillations

**Figure 7:**
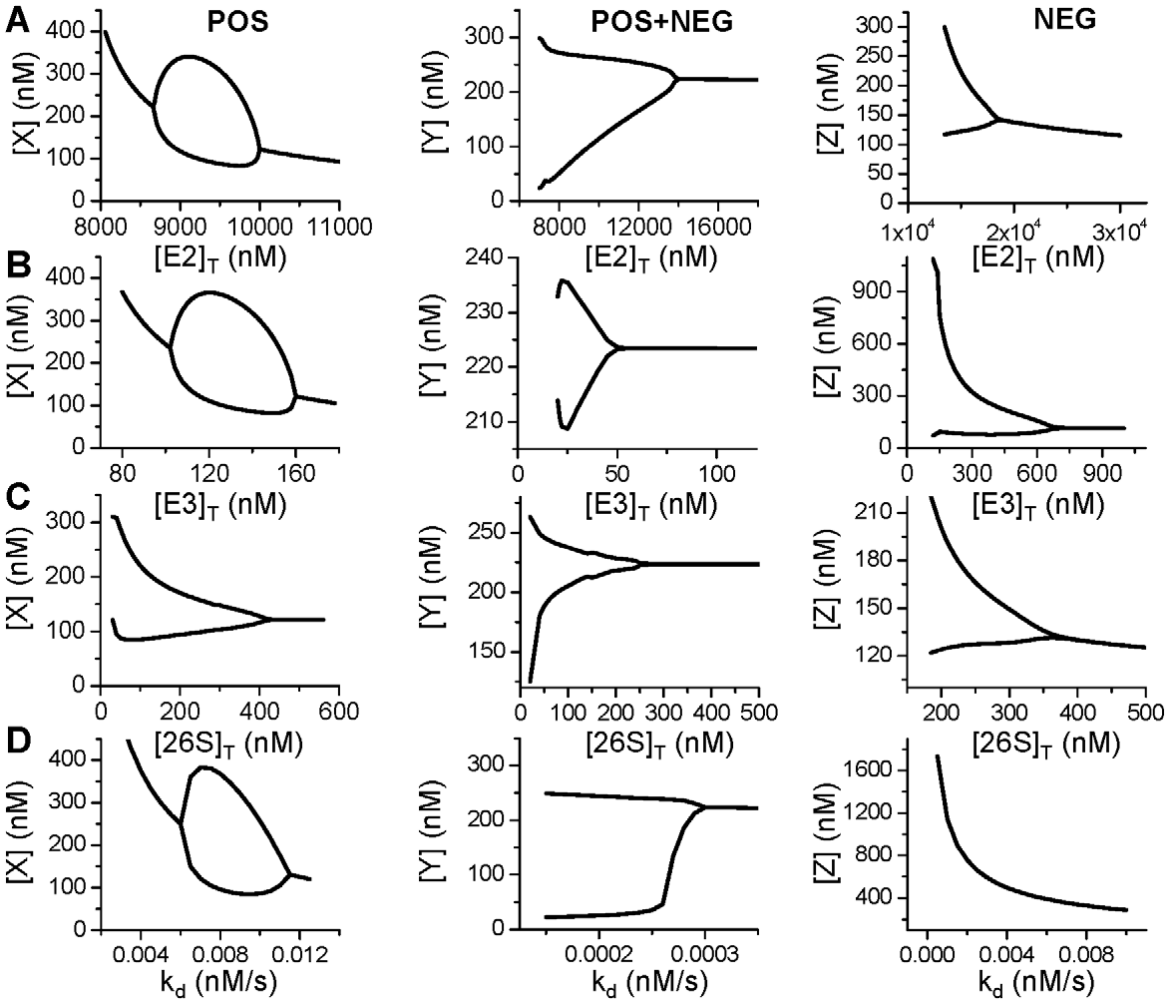
Effects of the UPS proteins on oscillations. **A**. Bifurcation diagrams showing the effects of E2 on oscillations from the three different mechanisms. Plotted are maximum and minimum values of a substrate protein versus [E2]_T_. **B**. Same as A but for E3. **C**. Same as A but for 26S. **D**. Same as A but for linear degradation kinetics.

In the theoretical analysis above, the degradation kinetics is represented by simple functions. To study how a specific protein affects the oscillations of different mechanisms, we use the detailed UPS model for the ubiquitination and degradation of the proteins in the three models of oscillations. The models were rescaled to reflect the real units of time and protein concentrations with the transformed equations presented in section of Methods and Materials. In Fig. 7, we show bifurcation diagrams for the three mechanisms of oscillations and for different UPS proteins, [E2]_T_ (Fig. 7A), [E3]_T_ (Fig. 7B), and [26S]_T_ (Fig. 7C). Since changing the concentrations of these proteins affects the maximum degradation rate, the dissociation constant, and the ubiquitination and deubiquitination time constants, how they affect the oscillations is not straightforward. For comparison, we also plot the bifurcation diagrams using linear degradation kinetics for each case (Fig. 7D).

For the positive feedback mediated oscillations (left panels in Fig. 7), decreasing either [E2]_T_ or [E3]_T_ first promotes oscillations and then suppresses oscillations (left panels of Fig. 7B and C). These bifurcation diagrams are very similar to the one for the linear degradation kinetics (left panel in Fig. 7D). As shown in Fig. 2, decreasing [E2]_T_ or [E3]_T_ decreases the maximum degradation rate α, indicating that reducing the degradation rate is the major cause of the bifurcation sequences. In the case of varying [26S]_T_, oscillations occur when [26S]_T_ is reduced to 400 nM, but decreasing [26S]_T_ has little effects on α and β until [26S]_T_ is small (<100 nM) during which α and β decreases as [26S]_T_ decreases. Note that the steady state is a constant between [26S]_T_=400 nM and [26S]_T_=600 nM, indicating no change in the degradation rate as [26S]_T_ is reduced from 600 nM to 400 nM. A possible cause of instability is the reduction in τ_ub_ as [26S]_T_ reduces (Fig. 3D), which agrees with the theoretical analysis that reducing the time delay of ubiquitination promotes instabilities.

For the positive-plus-negative feedback mediated oscillations (middle panels in Fig. 7), decreasing either [E2]_T_, [E3]_T_, or [26S]_T_ promotes oscillations, as in the case of linear degradation kinetics. As shown in the simple model, the degradation kinetics has only a small effect on the oscillations, therefore, the major effects of these proteins on oscillations are through their effects on altering the rate of degradation.

For the negative feedback mediated oscillations (right panels in Fig. 7), decreasing either [E2]_T_, [E3]_T_, or [26S]_T_ promotes oscillations until the degradation rate is too low (smaller than the synthesis rate) to maintain a finite equilibrium state. However, for the same Hill coefficient (*p*=4) of the negative feedback term, no oscillations can be seen in the linear degradation kinetics (right panel in Fig. 7D), indicating that the oscillations is due to the MM degradation kinetics.

**Figure 8:**
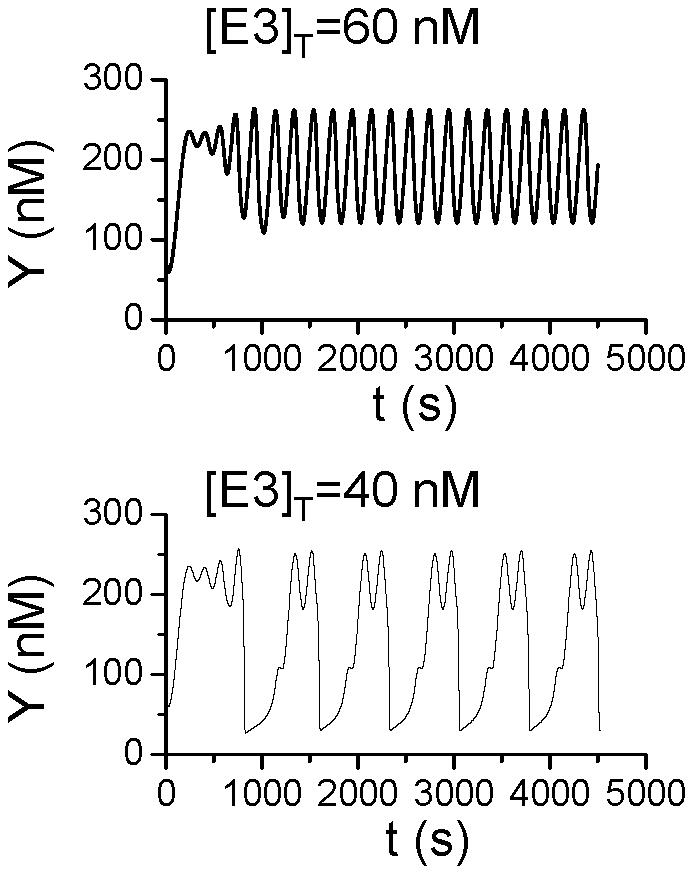
Complex oscillations caused by time delay in the UPS for positive-plus-negative feedback mediated oscillations. [Y] versus time for two different [E3]_T_. The plots are the same as Fig. 5B except that the detailed UPS model and Eq.12 are used.

Agreeing with the observations in the simple model, the time delay in ubiquitination and deubiquitination causes complex oscillations in the positive-plus-negative feedback mediated oscillations. Figure 8 shows two simulations when the simple degradation kinetics in Eq.5 was substituted by the detailed UPS model for two different [E3]_T_. When [E3]_T_=60 nM, the oscillations are regular, but when [E3]_T_ was reduced to 40 nM, complex oscillations occur to similar to the one shown in the simple model (Fig. 5B). As shown in Fig. 3C, reducing [E3]_T_ increases the time constant τ_ub_ and τ_Dub_, which agrees with the observations in the simple model that the complex oscillatory behavior is caused by the time delay in the UPS.

## Discussion

Protein degradation is known to be important for many biological functions and the major effect is to maintain a proper protein level for a normal biological function. However, the roles of the protein degradation kinetics have not been well understood. The study by Wong et al [18] shows that the degradation kinetics may play an important role in promoting oscillations and the study by Buchler et al [42] shows that the nonlinearity in protein degradation can be important for bistability of biological systems. In this study, we developed a detailed biochemical reaction model of protein ubiquitination and degradation based on a previous model using experimental data [23], and showed that the degradation kinetics mainly follows the MM kinetics. We then performed theoretical analyses in simplified models to show how MM kinetics of protein degradation promotes oscillations originating from different biochemical mechanisms, comparing these observations to those with linear degradation kinetics. We showed that the time delay occurring during ubiquitination and deubiquitnation always suppress instabilities but can promote complex oscillations. We also used the detailed model to study how the specific proteins in the UPS affect oscillations and showed that these effects could be explained using results from the theoretical analyses of the simple models.

Comparing with the previous studies on the effects of the protein degradation kinetics [18], [42], the novel aspects of the present study are as follows: 1) a detailed model of protein ubiquitination and degradation was developed to study the degradation kinetics; 2) a general theoretical analysis of the effects of the degradation kinetics on stability of the equilibrium state was performed for different mechanisms of oscillations, and compared with those of linear degradation kinetics in an unbiased manner; 3) with the detailed model and the theoretical results, one can study the impact of a specific protein in the UPS on the nonlinear dynamics of biochemical reactions. The implications of our present study to biological oscillations are as follows—the nonlinear degradation kinetics and time delay can promote: 1) Hopf instability of the equilibrium state for oscillations; 2) larger amplitude and lower frequency oscillations; and 3) complex oscillations. These analyses offer new mechanistic insights into the effect of individual protein components of the UPS—specifically, E2, E3 and 26S—on oscillations. However, as biological systems are regulated by complex protein networks [43], [44] and are across many scales [45], [46], conclusions from a simplified model need to be cautiously interpreted and eventually validated in experimental studies. Moreover, since almost all proteins undergo ubiquitination and degradation, how a specific protein in the UPS affects biological oscillations needs to be studied in the context of the whole network, and for different classes of substrate proteins. Finally, although we used a detailed and experimentally-based model of protein ubiquitination, the model of proteasome is simple. As shown in other modeling studies [47], [48], the proteasome kinetics may be also nonlinear, which may introduce more complex nonlinearity into the protein degradation kinetics and is worth studying in future works. Nevertheless, our present study shows that besides the rate of degradation, its kinetics might play important roles in biological functions under normal and diseased conditions. In addition, our study also shows that in mathematical models of biochemical reactions, instead of using the widely used linear kinetics, one needs to consider using protein degradation with proper kinetics that more accurately capture the biological features of the UPS.

## Materials and Methods

The detailed mathematical model of UPS and protein degradation was developed based on the reaction scheme in Fig. 1 following the law of mass action. The differential equations and the control parameters are presented in Table 1. The differential equations are solved using a fourth-order Runge-Kutta method.

When the detailed protein ubiquitination and degradation model was used in the three models of oscillation, the variables in these models need to be rescaled to the real units of time and protein concentrations. We rescaled the concentrations by 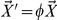 (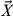 and 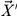 are the vectors of the protein concentrations) and time by 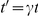.

For the positive feedback model (Eq.3), the rescaled equations are:
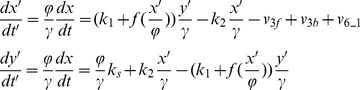
(10)where we used *ϕ*=150, and *γ*=200. Note that *v*_3f_ is the rate of protein X binding with E3 for ubiquitination and degradation. For 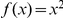, then 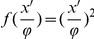. *v*_3f_, *v*_3b_, and *v*_6_1_ are the rates shown in Table 1 with substrate S substituted by protein X.

For the Goodwin model (Eq.7), we assume that the variables *x*, *y*, and *z* use the common UPS, and therefore, the total amount of [E1], [E2], [E3], [ub], and [26S] are three times as the amount used in the positive feedback model. We rescale Eq.7 to:
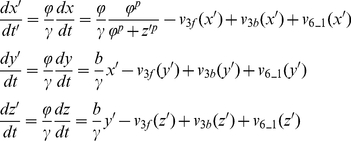
(11)where *ϕ*=150, *γ*=0.8, *v*_3f_, *v*_3b_, and *v*_6_1_ are the same as in Table 1 with substrate S substituted by protein X, Y, and Z, respectively.

**Figure 9:**
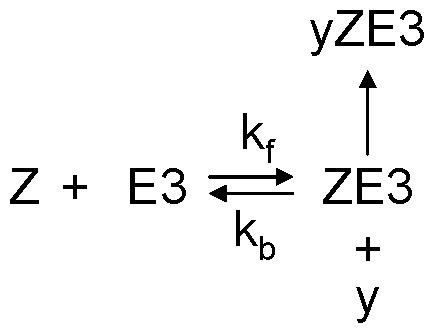
Reaction scheme for protein Y binding with E3 in the UPS and protein Z in the positive-plus-negative feedback model.

For the positive-plus-negative feedback model, we modified the binding of protein Y to E3 by also binding with protein Z as the reaction scheme shown in Fig. 9. The differential equations (Eq. 6) are rescaled to:
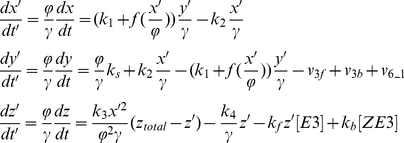
(12)We used *ϕ*=50, *γ*=20, *k*_f_=1 (nM s)^−1^, *k*_b_=0.5 s^−1^, and *z*_total_=1000 nM. *v*_3f_, *v*_3b_, and *v*_6_1_ are the same as in Table 1.
